# Analyzing the structure and function of neuronal circuits in zebrafish

**DOI:** 10.3389/fncir.2013.00071

**Published:** 2013-04-23

**Authors:** Rainer W. Friedrich, Christel Genoud, Adrian A. Wanner

**Affiliations:** Friedrich Miescher Institute for Biomedical ResearchBasel, Switzerland

**Keywords:** zebrafish, neuronal circuit, reconstruction, activity pattern, olfactory system

## Abstract

The clever choice of animal models has been instrumental for many breakthrough discoveries in life sciences. One of the outstanding challenges in neuroscience is the in-depth analysis of neuronal circuits to understand how interactions between large numbers of neurons give rise to the computational power of the brain. A promising model organism to address this challenge is the zebrafish, not only because it is cheap, transparent and accessible to sophisticated genetic manipulations but also because it offers unique advantages for quantitative analyses of circuit structure and function. One of the most important advantages of zebrafish is its small brain size, both at larval and adult stages. Small brains enable exhaustive measurements of neuronal activity patterns by optical imaging and facilitate large-scale reconstructions of wiring diagrams by electron microscopic approaches. Such information is important, and probably essential, to obtain mechanistic insights into neuronal computations underlying higher brain functions and dysfunctions. This review provides a brief overview over current methods and motivations for dense reconstructions of neuronal activity and connectivity patterns. It then discusses selective advantages of zebrafish and provides examples how these advantages are exploited to study neuronal computations in the olfactory bulb.

During the last century, a series of seminal discoveries demonstrated that brains are constructed modularly from distinct types of neurons, that information is transmitted by discrete action potentials, that electrical signals are generated and shaped by a plethora of ion channels, and that signals are passed and modulated through synapses (Albright et al., 2000). Many of these phenomena are now understood, in principle, at the molecular and biophysical level. Additional results provided detailed anatomical descriptions of the brain, uncovered mechanisms governing brain development, and revealed the engagement of defined brain regions in perceptual and cognitive tasks. Nevertheless, for many brain functions it is still unclear how they emerge from the biophysical properties of neurons and their interactions. Important elementary computations underlying higher brain functions are performed by subsets of neurons – neuronal circuits – that are typically defined as anatomically distinct networks of 10^2^ – 10^7^ neurons in vertebrates. Because circuit-level computations depend on dynamic interactions between large numbers of neurons, they cannot be fully analyzed by studying one neuron at a time. Rather, understanding neuronal circuit function also requires quantitative analyses of activity patterns across neuronal populations and rigorous analyses of network connectivity. Since neuronal circuits are stunningly complex even in comparison to other biological systems, a profound understanding of neuronal circuits is an enormous task. However, without such an understanding, key aspects of the brain remain elusive, and the rational design of treatments for psychiatric and neurological disorders is severely hampered. Quantitative analyses of neuronal circuit structure and function therefore present an outstanding scientific challenge, not only for neuroscience but also for other fields such as engineering and theoretical disciplines.

## ANALYSIS OF NEURONAL CIRCUITS: METHODS AND MODEL SYSTEMS

Over the last decade, technological developments have opened fundamentally new opportunities to study neuronal circuits. These include sophisticated molecular approaches to identify, label and manipulate specific types of neurons in the brain, quantitative paradigms to study behavior, advances in extracellular recording techniques to measure action potential firing of multiple neurons in behaving animals, and important developments in intracellular recording methods (Luo et al., 2008; Scanziani and Hausser, 2009). In addition, three technologies are currently changing the landscape of neuroscience research. First, multiphoton calcium imaging can visualize activity patterns across large numbers of neurons with single-neuron spatial resolution and a temporal resolution between a few milliseconds and approximately a second (Denk and Svoboda, 1997; Kerr and Denk, 2008). Although multiphoton microscopy was first described more than 20 years ago (Denk et al., 1990), the technique became widely used only recently, partly because optical know-how has spread within the neuroscience community and because microscopes with good performance can now be obtained commercially. In parallel, genetically encoded calcium indicators were optimized to the point that they reliably report the occurrence of one or a few action potentials with sufficient signal-to-noise ratio (Akerboom et al., 2012; Looger and Griesbeck, 2012). As a consequence, multiphoton calcium imaging is now used in many laboratories to measure neuronal activity across populations of neurons, providing direct insights into the function of neuronal circuits.

Second, opto- or pharmacogenetic tools have been developed to depolarize or hyperpolarize defined neurons by light or by specific chemical compounds, respectively. Neuronal activity can now be manipulated with unprecedented spatial, temporal and cell type specificity through the intersection of genetic targeting and optical stimulation (Bamann et al., 2010; Mattis et al., 2011; Yizhar et al., 2011; Zhang et al., 2011). When applied in behaving animals, opto- and pharmacogenetic manipulations can uncover causal relationships between the activity of identified neurons and behavioral outputs. Furthermore, opto- or pharmacogenetic tools can be used to perturb activity patterns within an active circuit, to impose specific neuronal activity patterns onto a population, and to up- or downregulate the activity of specific cell types. These approaches are extremely valuable for systematic analyses of functional circuit properties *in vitro* and *in vivo*.

Third, novel methods have been developed to analyze the connectivity between neurons in a circuit. Genetic labeling with combinations of fluorescent proteins permits light-microscopic tracing of multiple neurons within a tissue (Livet et al., 2007; Lichtman and Denk, 2011), and transsynaptic viral tracers can visualize neurons that are monosynaptically connected to one or a few target neurons (Wickersham et al., 2007; Luo et al., 2008). These approaches cannot, however, reconstruct the complete set of neuronal connections in most circuits. Currently, dense circuit reconstructions rely on morphological tracing of neurons and on the identification of their synaptic connections in image stacks. This approach requires high spatial resolution (~25 nm or better) throughout large volumes (often >100 μm in each dimension; Lichtman and Denk, 2011). In small volumes, nanometer resolution has been achieved by imaging of serial ultrathin sections in a transmission electron microscope (Harris et al., 2006) but this approach cannot easily be scaled up because it depends heavily on manual labor. Recently, methods for efficient ultrastructural imaging of large volumes have been developed that are based on the automated sectioning of a tissue block (Denk and Horstmann, 2004; Hayworth et al., 2006; Kasthuri et al., 2007; Helmstaedter et al., 2008; Knott et al., 2008; Briggman and Bock, 2012; Denk et al., 2012). In one approach, an automated tape-collecting ultramicrotome (ATUM) is used to cut sections at a thickness of <30 nm and collect them on a carbon-coated tape (Hayworth et al., 2006; Kasthuri et al., 2007; Tapia et al., 2012). Sections are then imaged in a scanning electron microscope (SEM). Other approaches section the tissue block inside the vacuum chamber of an SEM and take images of the block face, rather than the section, after each cut. Sections can be either cut by a diamond knife (SBEM), which achieves thicknesses <25 nm and offers a large field of view (> 1 mm), or milled by a focused ion beam (FIB-SEM), which achieves thicknesses down to 5 nm but in a smaller field of view (<80 μm; Denk and Horstmann, 2004; Helmstaedter et al., 2008; Knott et al., 2008; Briggman and Bock, 2012). An advantage of the ATUM approach is that sections are preserved, allowing for post-staining, repeated imaging, and parallel imaging in multiple microscopes. Block face methods discard sections but minimize image registration problems, achieve thinner cutting, and have been reported repeatedly to cut thousands of sections without a single loss (Denk and Horstmann, 2004; Briggman et al., 2011). Although 3D electron microscopy and the associated sample preparation methods are demanding, the rate-limiting step for the reconstruction of entire circuits is usually data analysis, i.e., the tracing of neurons and the identification of synapses in stacks of EM images (Helmstaedter et al., 2008). The current gold standard for the reliable reconstruction of neurons is manual tracing (Helmstaedter et al., 2011), making the dense reconstruction of large circuits an enormous task. However, as connectivity imposes hard constraints on the exchange of information between neurons, solid and comprehensive information about a circuit‘s wiring diagram is highly valuable and, in many cases, likely to be necessary to understand how a circuit computes (Briggman and Bock, 2012; Denk et al., 2012). Reconstructing wiring diagrams of neuronal circuits is therefore a critical challenge in systems neuroscience.

To exploit the full potential of novel methods it is important to apply them in appropriate model systems. History shows that the selection of animal models such as *Drosophila*, mice, *C. elegans* or *Aplysia* has been critical for breakthrough discoveries, much like the development of novel technologies. Because many approaches to neuronal circuits rely on genetically encoded probes there is a strong incentive to choose a species for which advanced molecular and transgenic methods are established. Among invertebrates, obvious candidates are *C. elegans* and *Drosophila*. Some principles of information processing in other species can, however, not be addressed in *C. elegans*. Moreover, electrophysiological recordings are difficult, and the behavioral repertoire is limited. Many results obtained in *Drosophila* have been instructive and can be generalized to vertebrates. Interesting insights into general computational principles are likely to emerge from comparative studies of neuronal circuits that evolved independently but perform similar tasks in invertebrates and vertebrates. Some brain functions, however, are likely to differ between insects and vertebrates, as suggested by obvious differences in general brain anatomy and many other observations. It is thus desired to complement insect model systems with vertebrate models that offer similar experimental advantages.

The main genetic model systems among vertebrates are the mouse and the zebrafish. Driven by advances in genetic methods, the mouse has become popular in neuroscience and many important techniques were established for experiments *in vitro* and *in vivo*. However, detailed analyses of neuronal circuit structure and function are still presenting a major challenge. An important limitation of mice is often that only a small fraction of the neurons involved in a given computation can be recorded, reconstructed or manipulated experimentally. Zebrafish have less of a history in neuroscience although they have no obvious principal limitations. In fact, recent studies demonstrated that key approaches such as whole-cell recordings, multiphoton calcium imaging, and quantitative behavioral analyses can be applied very efficiently. Moreover, the spectrum of methods for genetic manipulations has been extended significantly, for example by introducing two-component expression systems such as the Gal4- and the Tet-systems, and by establishing approaches for the targeted mutation of genes (Scott et al., 2007; Zhu et al., 2009; Huang et al., 2011; Bedell et al., 2012). Importantly, valuable resources have been created within the growing community of zebrafish neuroscientists, including large collections of Gal4 driver lines to target genetically encoded probes to specific types of neurons (Scott et al., 2007; Baier and Scott, 2009; Kawakami et al., 2010). An ongoing effort at the Sanger Center is creating mutations in every gene within the next few years (http://www.sanger.ac.uk/Projects/D_rerio/zmp/). As a consequence, zebrafish now offer a broad spectrum of opportunities for neurophysiological and molecular experiments that shows no obvious shortcomings compared to mice. Currently, the main limitation of zebrafish may be the availability of quantitative assays for complex behaviors. This situation is unlikely to reflect a limited behavioral repertoire of zebrafish but may simply be due to the fact that zebrafish neuroethology is still at an early stage. Indeed, various studies have demonstrated that zebrafish and closely related species display complex behaviors including schoaling, territorial behavior, kin recognition, associative learning including trace conditioning, place preference learning, spatial navigation, and others (Prober et al., 2006; Rodriguez et al., 2006; Gerlach et al., 2008; Saverino and Gerlai, 2008; Agetsuma et al., 2010; Norton and Bally-Cuif, 2010; Arganda et al., 2012; Karnik and Gerlai, 2012). It is therefore likely that advanced and quantitative behavioral assays for zebrafish can and will be developed in the future to study higher brain functions. A main difference between zebrafish and mice is their brain size. The zebrafish brain is substantially smaller both in terms of physical size and in terms of the number of neurons. Since small brain size provides clear advantages for quantitative analyses of neuronal activity and connectivity patterns, zebrafish offer the possibility to study features of neuronal circuits that cannot easily be studied in mice, as discussed below. The zebrafish therefore offers unique advantages for quantitative studies of neuronal circuit structure and function.

## ZEBRAFISH AS A MODEL IN SYSTEMS NEUROSCIENCE: SIZE MATTERS

Originally, the zebrafish has been chosen as a model system for genetics and developmental biology. Based on pioneering work by Streisinger et al. (1981), a group of researches including Christiane Nüsslein-Volhard, Monte Westerfield, and many others established important resources and used zebrafish to analyze vertebrate development by large-scale mutagenesis screens (see issue 123 of Development, 1996). Some advantages of zebrafish for developmental genetics, such as their transparency at early developmental stages and their low cost, are also useful for systems neuroscience. Nevertheless, neurophysiology remained an exotic area of research in zebrafish for many years. Recently, however, zebrafish neuroscience started to boom, which may be due to two major reasons. First, pioneering studies demonstrated that advanced methods including electrophysiology, imaging of genetically encoded probes, and optogenetics, can be used and combined very efficiently in larval and adult zebrafish. Second, as quantitative analyses of neuronal circuits moved into the focus of neuroscience, a growing community of scientists becomes interested in projects that appear feasible in zebrafish but daunting in larger species. As a consequence, zebrafish neuroscience has attracted scientists with diverse backgrounds and has become a highly dynamic and stimulating field.

Some advantages of zebrafish for neuroscience are “convenient” rather than “essential.” For example, the transparency of zebrafish larvae is often considered an advantage because it allows for calcium imaging of neuronal activity patterns and for optogenetic manipulations of neurons without the need for surgical procedures (O’Malley et al., 1996; Baier and Scott, 2009; Wyart et al., 2009; Blumhagen et al., 2011; Ahrens et al., 2012; del Bene and Wyart, 2012; Ahrens and Keller, 2013; Portugues et al., 2013). In some cases, however, surgical procedures are no principal barrier to reach the scientific goal. Neuronal population activity in some brain areas of behaving rodents can, for example, be measured by multiphoton calcium imaging using head-fixation and a virtual environment (Dombeck et al., 2007) or using head-mounted miniature microscopes (Sawinski et al., 2009; Ghosh et al., 2011). Likewise, optogenetic manipulations can be performed without dramatic experimental limitations using implanted optical fibers (Yizhar et al., 2011). Transparency is therefore essential only under specific experimental constraints, for example when optical access is needed simultaneously at different locations or from different directions (Ahrens et al., 2012; Tomer et al., 2012; Ahrens and Keller, 2013).

Other advantages of zebrafish are more fundamental because they enable experiments that cannot be performed in other organisms using available technology. Often, these advantages are related to the small size of the zebrafish brain. Size is a basic, yet very important, property of a model organism because key steps in the analysis of neuronal circuits have size constraints. These are particularly obvious for the exhaustive measurements of neuronal activity patterns by multiphoton calcium imaging and for the reconstruction of wiring diagrams by 3D-EM. The zebrafish brain is only <0.5 mm thick and 1.5 mm long in larvae, and between 0.4 and 2 mm thick and about 4.5 mm long in adults (Wullimann et al., 1996). The total number of neurons is on the order of 10^5^ in larvae and 10^7^ in adults (Hill et al., 2003; Hinsch and Zupanc, 2007).

The small physical size of the zebrafish brain obviously facilitates optical access for measurements of neuronal activity patterns by multiphoton calcium imaging. However, physical brain size is not always a principal limitation for imaging neuronal activity patterns because gradient index lenses or other technical solutions can now provide access even to deep neurons in the rodent brain (Ghosh et al., 2011). Rather, the primary constraint on measurements of neuronal activity patterns is often the absolute number of neurons that can be sampled during the time available for an experiment. Many experiments, particularly those that involve behavior, cannot be extended beyond a few hours and require the repeated application of multiple stimuli, separated by resting periods. As a consequence, the number of neurons whose activity can be sampled is typically not larger than a few thousand, and often much smaller. This number may be increased by future developments of technologies such as selective plane illumination microscopy (Tomer et al., 2012; Ahrens and Keller, 2013). However, solutions for exhaustive sampling of circuits that contain millions of neurons will likely remain difficult or impossible in the near future. In zebrafish, however, homologous circuits usually consist of much fewer neurons than in mice. The olfactory bulb (OB), for example, contains only ~500 neurons in larval zebrafish and 20000 – 30000 neurons in adults (Mack-Bucher et al., 2007; Wiechert et al., 2010), as compared to ~10^6^ – 10^7^ neurons in adult mice. Zebrafish therefore allow for the sampling of neuronal activity across a large fraction of neurons in many brain areas.

Why is exhaustive sampling of neuronal activity patterns important? Some computations of neuronal circuits can indeed be studied by sparse sampling. In particular, sparse sampling is sufficient when responses are dense and when a computation can be explained by simple statistical properties of neuronal activity patterns. For example, responses of individual neurons in sensory brain areas are often scaled as a function of the mean input by an operation termed “normalization” (Carandini and Heeger, 2011). This operation has been studied in detail in the retina and primary visual cortex for responses to well-defined stimuli such as gratings of different orientation. Under these conditions, normalization can be analyzed by measuring a neuron’s orientation tuning and estimating the mean population activity from a small number of recordings. This is possible because the computation does not depend on the precise structure of the population activity but only on its mean. Other functions of neuronal circuits, however, cannot be analyzed rigorously by sparse sampling. Dense sampling can, for example, be required to define the state of a network, particularly when these states are not triggered by an external event but occur spontaneously. Generally, dense sampling becomes important when neuronal activity itself is sparse and when information processing depends on specific subsets of neurons. In higher visual areas, for example, some neurons respond selectively to objects such as specific faces. For many stimuli, salient responses that contain much of the information about an object will therefore be missed when the population is sampled sparsely. Furthermore, many computations in the brain cannot be uncovered by measuring only first-order statistical properties of neuronal activity or connectivity patterns. For example, it is assumed that information is stored in memory networks by strengthening and weakening of specific synapses, resulting in the stabilization of specific neuronal ensemble responses during memory recall (Marr, 1970, 1971; McNaughton and Morris, 1987). In theory, such a stabilization of neuronal ensembles can occur without a major change in the mean activity across the population. For example, it is possible that the activity of some neurons increases while the activity of other neurons decreases so that activity patterns are reorganized, rather than enhanced or suppressed as a whole. It may be expected that such a reorganization affects specific, presumably sparse, subsets of neurons while the activity of many other neurons is not strongly altered. Moreover, it is possible that changes in synaptic coupling manifest themselves in the correlation between the activity of multiple neurons. In these cases, global statistical properties of activity patterns are insufficient to fully understand the computation. Dense measurements and detailed neuron-by-neuron analyses of activity patterns may therefore be required for rigorous insights into some important neuronal computations. Circuits whose function depends on sparse activity and on the specific structure of activity patterns are probably common in vertebrates, e.g., in the cortex and cerebellum.

Small brain size also has obvious advantages for the reconstruction of wiring diagrams by 3D-EM. One reason why small tissue samples are desired is that the acquisition of EM image stacks is slow. This is, however, not a hard limitation because sectioning and imaging of relatively large samples (millimeters) is technically feasible and because faster imaging is likely to become possible in the future (Denk et al., 2012). Moreover, since many questions about circuit connectivity can be addressed by analyzing a small number of specimens, imaging times on the order of weeks, months or even years may be tolerated. The main size constraint on circuit reconstruction comes from the fact that the analysis of the data is extremely laborious. So far, the reconstruction of neurons has been performed manually by humans. The first, and so far the only, circuit for which an almost complete wiring diagram has been published is the nervous system of *C. elegans*, which consists of only 302 neurons (White et al., 1986; Chen et al., 2006; Varshney et al., 2011). Nevertheless, the reconstruction involved the labor of many humans over many years. More recently, large numbers of neurons in the mammalian retina have been reconstructed by humans who traced center lines (skeletons) of neurites using specialized, user-friendly software (Briggman et al., 2011; Helmstaedter et al., 2011). The tracing speeds obtained by this approach were on the order of 5–6 h/mm path length, not including error correction and synapse identification (Helmstaedter et al., 2011). The dense reconstruction of large circuits is therefore an enormous task considering that a cubic millimeter of cortical tissue contains approximately 4.5 km of neurites (Braitenberg and Schüz, 1998). Large-scale tracing of neurites is currently addressed by recruiting large cohorts of human tracers (“crowd-sourcing”) and by the development of automated reconstruction methods (Turaga et al., 2010; Ciresan et al., 2012). It is, however, likely that the exhaustive reconstruction of large circuits will remain a massive task for a considerable future. Without automated procedures that increase reconstruction speed by orders of magnitude it is expected that the sheer bulk of the task will make the reconstruction of many circuits impossible in practice. A small model system such as zebrafish can therefore provide major advantages.

Some of the reasons why dense reconstructions of wiring diagrams are important are closely related to the reasons why dense measurements of neuronal activity patterns are important. Sparse sampling of connections may be sufficient to understand neuronal computations that depend only on simple statistical features of the connectivity matrix. For example, to normalize the output of individual neurons as a function of the mean population activity, neurons have to receive a signal reflecting the mean population activity. This signal does not require specific connectivity between individual neurons but can be extracted by neurons receiving stochastic, and sufficiently dense, input from the network. The statistical properties of connectivity required to understand the essence of this computation – averaging – can thus be obtained by sparse probing of connections. Other computations, however, require more detailed knowledge of wiring diagrams. A recent study in the retina revealed that direction-selectivity of ganglion cells depends on synaptic input from specific subsets of starburst amacrine cells, which was revealed by reconstructions of multiple neurons within the same retinal tissue block (Briggman et al., 2011). Precise knowledge of connectivity is therefore important to understand the mechanistic basis of some computations even in the retina, where cell types and mean connectivity have been analyzed in more detail than in most other brain areas. Detailed and exhaustive analyses of neuron-by-neuron connectivity should be particularly important for neuronal circuits whose functions are shaped by experience. For example, it is assumed that the storage of information is accomplished by the strengthening or weakening of specific synaptic connections and, on longer timescales, by the elimination and formation of synaptic connections in a network. The reconstruction of the precise synaptic connectivity between many neurons would therefore provide a direct approach to analyze information storage by networks of neurons (Seung, 2009).

Dense reconstructions of wiring diagrams will immediately provide novel information about topological features of neuronal circuits such as reciprocal or circular connectivity, cliques of interconnected neurons and other structural “motifs.” This information is of central importance for computational modeling and theoretical approaches to neuronal circuit function. Obviously, wiring diagrams provide hard constraints for circuit models but, by themselves, are most likely insufficient to explain and predict the function of many circuits. Detailed wiring diagrams may therefore be necessary, but not necessarily sufficient, to understand how a circuit computes (Briggman and Bock, 2012; Denk et al., 2012). An important goal in the field is therefore to combine the reconstruction of wiring diagrams with functional studies of neurons or neuronal ensembles, an approach that was, for example, used to analyze direction-selective circuits in the retina (Briggman et al., 2011).

The small brain of zebrafish provides essential advantages for exhaustive measurements of neuronal activity patterns and the underlying connectivity. Below, we will briefly review recent studies from our own group that have exploited these advantages to study the structure and function of neuronal circuits in the OB, the first olfactory processing center in the brain.

## FUNCTIONAL AND STRUCTURAL ANALYSIS OF NEURONAL CIRCUITS IN THE OLFACTORY BULB OF ZEBRAFISH

The OB receives direct input from sensory neurons in the nose through an array of discrete neuropil structures, the glomeruli. Each glomerulus receives input from sensory neurons expressing the same odorant receptor. Individual odorant receptors can be activated by a spectrum of ligands, and each odorant activates a specific combination of odorant receptors. In the input layer of the OB, odors are therefore represented by a specific pattern of afferent activity across the array of glomeruli. In zebrafish, these odor-evoked input activity patterns have been visualized by voltage- or calcium-sensitive dye imaging of sensory axons (Friedrich and Korsching, 1997, 1998). Glomerular activity patterns are processed by a distributed network consisting of principal neurons, the mitral cells (MCs), and various types of local interneurons including granule cells, periglomerular cells and short axon cells. OB output is then conveyed by MCs to multiple higher brain areas.

Calcium imaging demonstrated that chemically similar amino acids, which are natural odorants for teleosts, activate specific, yet highly overlapping, combinations of glomeruli (Friedrich and Korsching, 1997). Activity patterns evoked by the same stimuli across MCs become more distinct during an odor response, as revealed by electrophysiological recordings and multiphoton calcium imaging (Friedrich and Laurent, 2001; Friedrich et al., 2004; Yaksi and Friedrich, 2006; Yaksi et al., 2007). Hence, neuronal circuits in the OB perform a pattern decorrelation, an elementary computation that can facilitate odor discrimination and autoassociative memory storage. This decorrelation was observed when responses from a substantial fraction of MCs were recorded. If the number of MCs in the analysis is reduced by removing MCs from the sample, pattern decorrelation became increasingly more difficult to detect. Hence, a sufficient density of sampling is required to observe this computation. This density has been achieved in the OB of adult zebrafish, which contains approximately 1500 MCs (Yaksi et al., 2007), but may be difficult to achieve in the OB of mice, which contains approximately 50000 MCs, distributed throughout a large volume.

A decorrelation of activity patterns appears useful when overlapping patterns represent different information but is counterproductive when overlapping patterns are noisy representations of the same stimulus. This conflict could be resolved if MC activity patterns are stable against small differences in the input but become decorrelated when differences exceed a certain range. To test this possibility, we “morphed” one odorant into a similar one through a series of intermediate mixtures with different concentration ratios and measured activity across large numbers of MCs by multiphoton calcium imaging. Morphing of the odor stimulus resulted in MC activity patterns that remained similar within certain ranges of the morphing series but became suddenly decorrelated at the transition between these stability ranges (Niessing and Friedrich, 2010). Hence, decorrelation divides the coding space of MCs into discrete, relatively stable regions that are separated by instable transition regions. This discontinuous decorrelation can act as a sensory filter and results in a discrete classification of odor representations. The potential number of stable regions is very large, implying that discretized MC activity patterns represent the stimulus space at high resolution. Further analysis showed that the decorrelation at transition points was mediated by coordinated response changes among small ensembles of MCs, rather than by shifts in the global network state (Niessing and Friedrich, 2010). Decorrelation is therefore mediated by distinct, small subsets of MCs, which explains why it is difficult to observe when only few neurons are analyzed. Hence, a detailed study of pattern decorrelation in the OB requires sufficiently dense sampling because the computation depends on sparse and specific subsets of neurons.

Computational modeling and theoretical analyses revealed that pattern decorrelation can emerge from thresholding, a generic operation performed by spiking neurons, and from sparse recurrent connectivity within the circuit (Wiechert et al., 2010). Abrupt transitions between output patterns might be created by connectivity among specific ensembles of neurons, although other mechanisms are also conceivable. A thorough analysis of the connectivity underlying pattern decorrelation may therefore require dense reconstruction of the circuit. Detailed knowledge of the wiring diagram is also expected to reveal other important structural features of the circuit. We therefore started to reconstruct neurons in the OB and their connections by SBEM and manual tracing. Because this is a considerable task we are currently applying this approach to the OB of larvae, rather than adult fish (Miyasaka et al., 2012).

**FIGURE 1:**
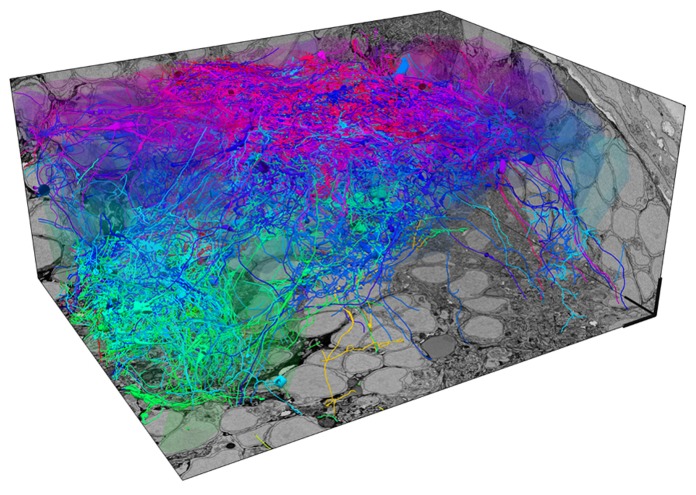
**Exhaustive reconstruction of neurons in the olfactory bulb of a zebrafish larva by serial block face scanning EM (SBEM).** Shown are skeleton reconstructions of 137 neurons associated with different developing glomeruli (protoglomeruli) in the OB of a zebrafish larva (four days post-fertilization). Somata are transparent to enhance visibility of neurites. Each neuron has been manually reconstructed by three human tracers. Skeletons represent the consensus of the three reconstructions for each neuron. The diameter of skeletons represents the variation in the redundant reconstructions, providing a rough estimate of the neurite’s diameter. Neurons are color-coded according to their soma location along the z-axis. Scale bars: 5 μm.

In larvae expressing a genetically encoded calcium indicator in almost all neurons, we first measure responses of up to 50% of all neurons in one OB to different odors by multiphoton microscopy. After fixation, staining and embedding of the sample, a stack of EM images covering the same OB is then acquired by SBEM with a voxel size of approximately 10 nm^3^ × 10 nm^3^ × 25 nm^3^. Image acquisition takes 2–3 weeks and the total volume of the stack is approximately 90 μm^3^ × 120 μm^3^ × 70 μm^3^. However, the subvolume that is filled by neurites and presents the major challenge for reconstruction is substantially smaller because a large fraction of the total volume is occupied by somata. In one OB, we have so far manually reconstructed skeletons of approximately 75% of all neurons with the help of external tracers (**Figure 1**). Each neuron has been reconstructed multiple times by different individuals to detect, analyze and correct tracing errors. Although the quantitative evaluation is still ongoing, preliminary results indicate that the reliability of reconstructions is high. Most discrepancies between different tracings of the same neurons appear to be due to individual mistakes, for example when a tracer missed a branch point. Such errors are easy to detect and correct. Disagreement originating from ambiguities in the data, which may be caused by insufficient resolution or contrast, appears to be very rare. Since the staining methods used in this study generate contrast of extra- and intracellular membranes, synapses can be identified visually in the EM images. Quantitative comparisons with EM images obtained at higher resolution are underway to determine the reliability of synapse identification in stacks obtained by SBEM. Although the manual reconstruction of an entire OB is a substantial task, it can be accomplished with the help of a limited number of external tracers (<50) within a reasonable time frame (<1 year). Assuming that reconstruction time scales with volume, the reconstruction of all neurons in the OB of a mouse by the same approach would take many kiloyears.

The goal of this study is to reconstruct all neurons within the OB, identify most of their synaptic connections, and relate the resulting connectivity matrix to the functional response properties of neurons measured by multiphoton calcium imaging. Such a dense reconstruction of activity and connectivity patterns in a complete circuit is expected to provide novel insights into circuit function that may be difficult, or even impossible, to obtain by other approaches.

## CONCLUDING REMARKS

The zebrafish is becoming a popular model for studying the structure and function of neuronal circuits because it presents a variety of advantages over other animal models. Some of these advantages are useful, although not essential, while others enable experiments that are difficult or impossible to perform in other genetic model organisms. A key advantage of zebrafish, both at larval and adult stages, is its small size. Small brains are particularly useful and, in some cases, essential for quantitative and exhaustive studies of neuronal activity and connectivity patterns. As such analyses are a major bottleneck in the mechanistic analysis of many neuronal computations, zebrafish have the potential to promote true breakthrough discoveries in systems neuroscience. Moreover, ongoing efforts are establishing zebrafish models for various neurological, psychiatric and other diseases. Zebrafish also offer the opportunity to perform large-scale screens not only of mutant phenotypes, but also of small molecule effects on behavior and potentially other phenomena (Kokel et al., 2010, 2013; Rihel et al., 2010). It may therefore be expected that zebrafish will also become an interesting model system to take studies of neuronal circuits into the domain of translational research.

## Conflict of Interest Statement

The authors declare that the research was conducted in the absence of any commercial or financial relationships that could be construed as a potential conflict of interest.
